# Prions are the greatest protein misfolding problem, and yeast has several solutions

**DOI:** 10.1371/journal.ppat.1011333

**Published:** 2023-05-04

**Authors:** Reed B. Wickner, Herman K. Edskes, Songsong Wu, Kristen Gregg

**Affiliations:** Laboratory of Biochemistry and Genetics, National Institute of Diabetes and Digestive and Kidney Diseases, National Institutes of Health, Bethesda, Maryland, United States of America; Stanford University, UNITED STATES

“Prion” means “infectious protein,” with no nucleic acid required for transmission. Most prions involve self-propagating misfolding, usually amyloid (a filamentous polymer of a single protein rich in β-sheet). Prions are only one kind of misfolded protein, but they pose a much greater danger to the individual or species than other forms of misfolding because they are infectious. When a non-prion protein misfolds, it is only a problem for that molecule. A single event of prion formation can conceivably eliminate an individual, a population of a species, or even the entire species. This results in strong selection pressure for the cells/animals that have defenses against prions. Yeast anti-prion systems lower prion generation, cure nearly all prions that do arise, block prion infection, and mitigate prion pathology (Fig 1).

Human prion diseases include not only the various forms of classic Creutzfeldt–Jacob disease (transmissible spongiform encephalopathies) based on amyloid of PrP, but as recently found, the common amyloidoses Alzheimer’s disease, Parkinson’s disease, amyotrophic lateral sclerosis, and type 2 diabetes can occasionally be infectious and all have prominent prion-like features (e.g., [1]).

[PSI+] and [URE3] are cytoplasmic genetic elements [2,3] later found to be prions of Sup35p and Ure2p, respectively, of *Saccharomyces cerevisiae* ([4]; reviewed in [5]). Sup35p is a subunit of the translation termination factor, and [PSI+] cells show partial read-through of termination codons due to reduced amounts of normal Sup35. Ure2p negatively regulates expression of enzymes and transporters needed for catabolism of poor nitrogen sources, and in [URE3] cells, such genes are expressed in spite of the presence of a good nitrogen source. [PSI+], [URE3], and other yeast prions behave as non-chromosomal genes, and, like mammalian prions, yeast prions show the “prion strain” or “variant” phenomenon, in which a single peptide chain can form any of a large number of biologically (and structurally) distinct prions, each of which is rather stably self-propagating.

The similarity of yeast prions to the human prions/amyloidoses makes them an ideal model for exploring how the cell handles such disorders. Infectious amyloids of Sup35p and Ure2p are in-register parallel folded β-sheet structures [6,7], the same architecture as fully infectious amyloid of PrP [8], Aβ amyloid [9], and most human amyloids. The in-register parallel folded β-sheet architecture explains the ability of prions to template their conformation and, thus, the heritability and infectivity of prion strain/variant properties [5].

The [PSI+] and [URE3] prions are detrimental to yeast as shown by the high frequency of lethal and toxic variants [10], the rare occurrence of these prions in wild strains [11], and other considerations [5]. Thus, it is not surprising that yeast has an array of systems to defend against prions. Here, we briefly review the anti-prion systems and what is known of their mechanism of action (Fig 1).

**Fig 1:**
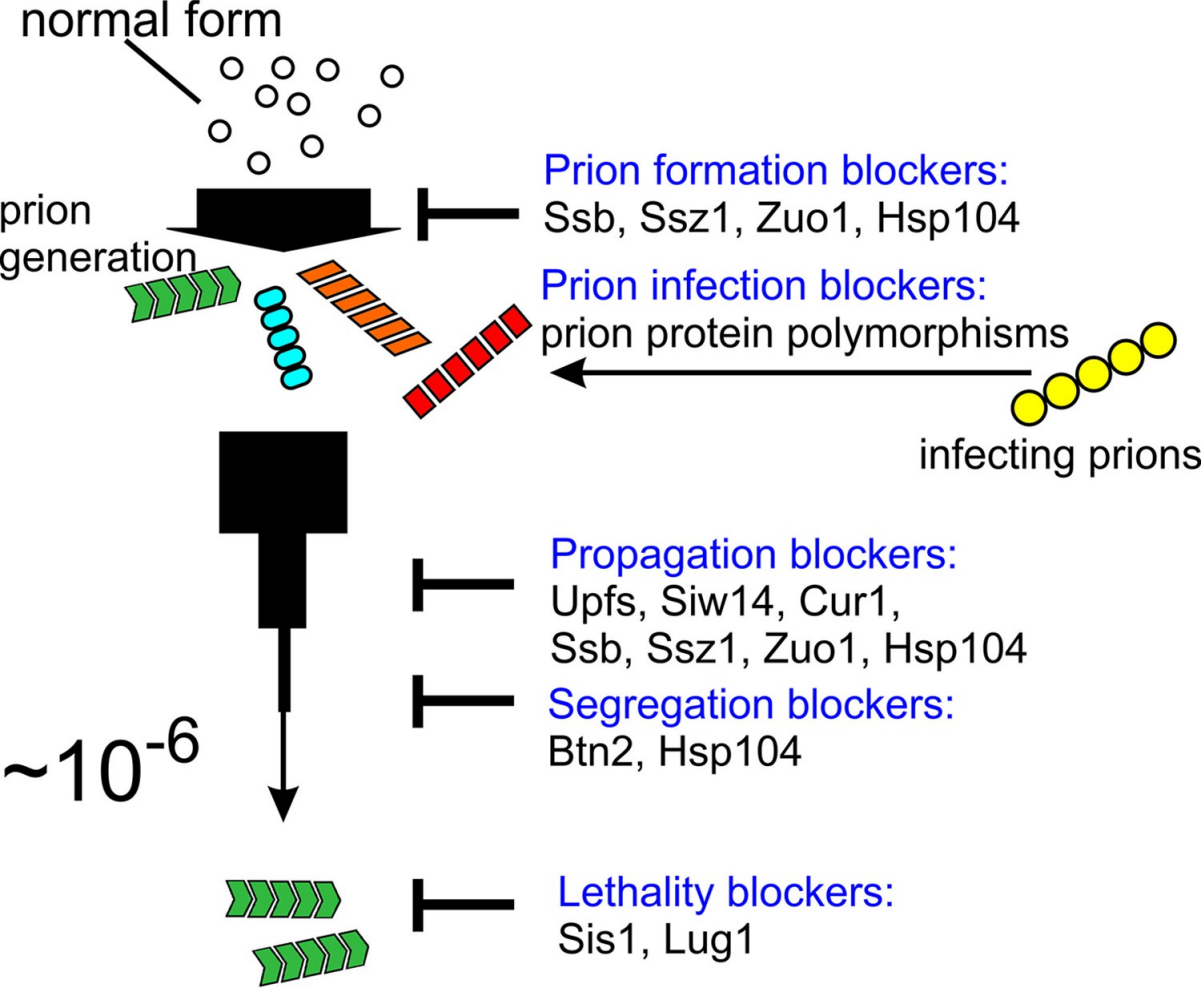
Anti–prion systems block infection, generation, segregation, propagation, and pathogenic effects of most prions.

## Ribosome-associated chaperones

Chernoff was the first to detect an anti-prion activity, showing that Ssb1/2, nearly identical Hsp70 –family ribosome-associated chaperones known to insure proper folding of nascent proteins, inhibit the formation of the [PSI+] prion [12]. He properly interpreted *ssb1Δ ssb2Δ* as mutator mutations, since [PSI+] and the other yeast prions are genes. Ssz1p (another Hsp70) and Zuo1p (an Hsp40) are associated with Ssb on the ribosomes, and *ssz1*, *zuo1*, and *ssb1/2* mutants each show [PSI+] generation frequency elevated 10- to 15-fold [12–14]. Over half of the [PSI+] prions generated in each of these mutants is cured by restoration of the mutant gene, expressed at its normal level [15]. These results show that the ribosome-associated chaperones act on both prion generation and on prion propagation. The role of these proteins in proper folding of nascent proteins would suggest that all prions might be similarly affected, but no effect on [URE3] was detected [15].

The Ssb proteins’ association with the ribosome is mediated in part by Zuo1p and Ssz1p, as mutants in either results in increased soluble Ssb1/2p. Elevated Ssb1/2p cures some [PSI+] variants, apparently by competing with the cytoplasmic Ssa family Hsp70s [13,16], but this must be a distinct activity from the anti-prion activities of Ssb1/2p, Zuo1p, and Ssz1p. For example, restoring normal levels of Zuo1p to a *zuo1Δ* mutant decreases the amount of soluble Ssb but cures most variants of [PSI+] that arose in the mutant.

## One Hsp104 activity has an anti-prion effect

Hsp104, acting with Hsp70s and Hsp40s, extract single molecules from an amyloid filament, thereby breaking the filament, creating new growing points, and thus replicating the prion [17–20]. However, on overproduction, Hsp104 cures [PSI+] and, less efficiently, [URE3] [17]. Mutants in the Hsp104 N-terminus (e.g., *hsp104T160M*) impair the curing activity but leave the prion propagation activity intact [21]. In *hsp104T160M* mutants, [PSI+] arises at 13-fold the normal rate, due to a failure to cure [PSI+] variants that are destabilized by normal levels of the normal Hsp104, and an increased frequency of generation of variants stable in a wild-type strain [22]. Thus, at normal levels, Hsp104 limits prion generation and cures a majority of the [PSI+] prions arising.

## Upf1, 2, 3 anti-prion effect on [PSI+]

An mRNA with a premature stop codon is degraded more rapidly than the normal mRNA, a process called nonsense-mediated decay and mediated by Upf1, 2, and 3 (reviewed in [23]). Deletion of *UPF1*, *UPF2*, or *UPF3* results in an approximately 15-fold increase in the appearance of [PSI+], with most of the prions appearing in the mutants being cured by restoration of normal levels of the Upf proteins [24]. Upf1 associates with Sup35 amyloid and blocks amyloid formation in vitro [24]. The Upf proteins are normally located on the ribosome in a complex with Sup35, and this association is responsible for the inhibition of prion generation and the curing activity [24]. It seems likely that, in general, normal protein interactions compete with or inhibit abnormal protein interactions, of which amyloid formation is one example.

## Inositol polyphosphates affect prion propagation

A screen for anti-[PSI+] genes revealed that Siw14, a pyrophosphatase specific for 5-diphosphoinositol pentakisphosphate (5PP-IP5), prevents the propagation of some [PSI+] variants, presumably because they require the higher 5PP-IP5 levels found in *siw14* mutants [25]. Most [PSI+] prions require one of the highly phosphorylated soluble inositol species, IP6, 5PP-IP5, or 5PP-IP4, but the mechanism of these effects remains obscure.

## Normal levels of Btn2 and Cur1 cure most [URE3] prions

Overproduction of Btn2 or its paralog Cur1 cure all [URE3] variants tested, but normal levels of these proteins cure a large majority of [URE3] prions formed in their absence [26,27]. Btn2 cures [URE3] by collecting Ure2 amyloid filaments at one place in the cell so that daughter cells are more likely to be prion free [26]. Unlike Btn2, Cur1 is not colocalized with the prion amyloid in the course of curing, and Cur1’s mechanism of action is as yet unknown. Proteasome impairment results in approximately 200-fold elevation of Btn2 and Cur1, thereby curing [URE3] [28]. Overwhelmed proteasomes may automatically invoke Btn2 sequestrase as a backup mechanism to deal with cellular stress.

## Ribosome-associated chaperones, Upf proteins, and Hsp104 act independently and lower prion appearance approximately 5,000-fold

In an *hsp104T160M ssz1*Δ *upf3*Δ strain, [PSI+] arises at up to 5,000-fold the normal frequency. Most of the prions arising are cured by restoration of any one of the defective genes, showing that they act independently and are 3 separate systems [29]. This also indicates that there are many prion variants that arise but are immediately cured. Among prions arising in the triple mutant are those that are stable in a normal host, and these arise at 25- to 100-fold the normal rate (not the approximately 5,000-fold overall rate) showing that prion generation is also affected by these systems.

## Btn2 and Cur1 are anti-[URE3] but pro-[PSI+]

Although Btn2 and Cur1 are clearly anti-[URE3] (see above), cure an artificial prion [30], and Btn2 collects non-prion aggregates [31], deletion of either gene dramatically reduces [PSI+] appearance and propagation [29], and almost no variants of [PSI+] are cured by Btn2 or Cur1 overproduction [32]. This difference was attributed to the much higher stability of [URE3] amyloid and the much higher propagon number of [PSI+] prions. As expected from its “sequestrase” activity, Btn2 cures [URE3] variants with low propagon number but only partly reduces the propagon number of high seed number prions [26,27]. Btn2 collects non-prion aggregates and may collect misfolded Sup35 molecules facilitating prion formation.

## Sis1 attenuates [PSI+] pathology

Although most [PSI+] prions are lethal or highly toxic, even the most mild [PSI+] can become toxic when Sis1 is C-terminally deleted. This Sis1JGF (retaining only the N-terminal J-domain (homologous to *E*. *coli dnaJ*) and the adjacent Gly-Phe–rich domain) performs the protein’s essential function for the cell and can propagate [PSI+], but strong [PSI+] variants are lethal because they soak up too much of the Sup35 [33].

## Lug1 lets [URE3] strains grow

A transposon mutagenesis screen for an inhibitor of [URE3] toxicity revealed that, without Lug1 [URE3], cells grow poorly or not at all [34]. Lug1 is an F-box protein, a substrate-specifying subunit of a cullin ubiquitin ligase, but the substrates that it specifies are not yet known.

## Perspectives

Misfolding of a few molecules of a protein is a minor event in the cell’s life, but if these few molecules form a prion amyloid, a potentially lethal, infectious process is initiated. Yeast has systems that block prion infection, inhibit prion generation, constrain prion propagation and segregation, and reduce the pathogenicity of those prions escaping these systems. We suggest that the discovery of multiple yeast anti-prion systems will facilitate the search for mammalian anti-prion systems. As we manipulate the induced and innate immune systems to deal with viral, bacterial, and parasitic diseases, anti-prion systems promise to afford relief from the now largely intractable prion/amyloid diseases that are widespread in our aging populations.
